# COVID-19 Vaccine Hesitancy in a Representative Education Sector Population in Qatar

**DOI:** 10.3390/vaccines9060665

**Published:** 2021-06-18

**Authors:** Reem Al-Mulla, Marawan Abu-Madi, Qusai M. Talafha, Reema F. Tayyem, Atiyeh M. Abdallah

**Affiliations:** 1 Department of Biomedical Sciences, College of Health Sciences, QU-Health, Doha 2713, Qatar; ra1305908@student.qu.edu.qa (R.A.-M.); abumadi@qu.edu.qa (M.A.-M.); 2 Biomedical and Pharmaceutical Research Unit, QU-Health, Qatar University, Doha 2713, Qatar; qusai.talafha@hu.edu.jo; 3 Department of Economics, Faculty of Economics and Administrative Sciences, The Hashemite University, Zarqa 330127, Jordan; 4 Department of Nutrition, College of Health Sciences, QU-Health, Doha 2713, Qatar; reema.tayyem@qu.edu.qa

**Keywords:** COVID-19, vaccine, hesitancy, Qatar, education sector, post vaccine administration program

## Abstract

Even though vaccination programs have now started in earnest across the globe and in Qatar, vaccine hesitancy remains a barrier to effectively tackling the pandemic. Many factors influence willingness to take vaccines including safety, efficacy, and side effects. Given their proximity to research and education, university students and employees represent an interesting cohort in which to investigate vaccine hesitancy. The aim of this study was to assess the attitudes of Qatar University employees and students towards the COVID-19 vaccine. In total, 231 employees and 231 students participated in an online cross-sectional study in February 2021. Of the sample, 62.6% were willing to take a vaccine against COVID-19. Participants with or taking postgraduate degrees were more willing to take the vaccine compared to participants with or taking a diploma or bachelor’s degree (*p* < 0.001). Males had a higher rate of vaccine acceptance (*p* < 0.001). In the group that regarded flu vaccination as important, 13% were unwilling to take COVID-19 vaccine. There were no associations between willingness to vaccinate and vaccine/virus knowledge and social media use. Participants showed a high level of concern regarding vaccine side effects in themselves or their children. Two-thirds agreed or strongly agreed that they would take the vaccine if it was mandatory for international travel. Our participants were neutral to the origin of vaccine development. These findings, which represent data collected after the start of the national vaccination program, show that vaccine hesitancy persists in the Qatari population and that some groups, such as undergraduate students, could benefit from specific, targeted public health campaigns.

## 1. Introduction

A number of vaccines to protect against COVID-19 have now been developed and distributed in the global fight against the pandemic. However, this effort has encountered an old and persistent enemy of public health, namely vaccine hesitancy. Vaccine hesitancy is a complex global issue listed as one of the top ten threats to global health in 2019 by the World Health Organization (WHO). In the online era, anti-vaccine activists and groups use social media and online campaigns to spread the erroneous belief that vaccinations are fundamentally unsafe [1]. As a result, communities have gone from being concerned about the spread of disease to being concerned about vaccine safety. Most parts of the world have faced two or three waves of COVID-19 infections with different virus strains. Moreover, countries that initially successfully controlled viral spread saw outbreaks return as restriction and control measure were eased. Herd immunity is a critical component of controlling infectious diseases; however, to achieve it, approximately 70% of the population must be vaccinated [2]. Therefore, as public uptake of the vaccine decreases, it becomes more difficult to achieve herd immunity. Several studies have examined vaccine hesitancy and acceptance since the start of the COVID-19 pandemic, even before the successful vaccine development and distribution, and a trend analysis of 126 surveys performed during March to October 2020 showed a worrying decline in vaccine acceptance from 70% in March to 50% in October [3].

The causes behind decisions to delay or refuse vaccinations are complex and vary widely. The fast-tracking of vaccine development and commercial interests of the pharmaceutical industry have further intensified public anxieties and could compromise acceptance [4]. The efficacy, side effects, and composition of vaccines are all important factors driving decisions to reject vaccination. The year 1998 saw a cornerstone moment in vaccine hesitancy when the Wakefield article published in The Lancet reported an association between autism and the triple MMR vaccine (a vaccine against measles, mumps, and rubella given to young children) [5]. This study resulted in a decrease in childhood vaccination in the UK to 80% [6]. In 2010, the study was retracted by the journal after they discovered fraudulent behavior and multiple undeclared conflicts of interest, including in research funding [7].

Defining vaccine hesitancy as a delay in acceptance or refusal of vaccination despite availability, vaccine hesitancy is common in all parts of the world and even affects all professional sectors, including healthcare and education. Studies of healthcare workers have shown high levels of vaccine rejection [8], even in physicians. In 2015, a French physician started a petition against vaccination, which received over one million signatures [9]. Physicians from Belgium, Austria, and other countries have publicly denounced vaccination [8]. Similarly, surveys have shown high levels of resistance to vaccination in academics. A recent study of medical students showed that 23% of participants were unwilling to take COVID-19 vaccine upon FDA approval [10].

University students and employees represent an interesting cohort to investigate vaccine hesitancy, as they are usually considered educated, broad-minded, and understanding of the threat posed to humans by infectious diseases. Therefore, the aim of this study was to examine COVID-19 vaccine acceptance and its determinants in Qatar University (QU) students and employees. Our sample was not designed to be representative of the entire Qatari population, rather it was a convenience sample to help understand the determinants of COVID-19 vaccine hesitancy in the education sector in Qatar; indeed, over 60% of employees at QU have an international background, mostly from Europe, America, and Australia, and over 50% of students are international students. Understanding attitudes toward COVID-19 vaccine in this unique educational setting and with international participants would therefore be of particular interest for enhancing vaccination uptake in this and similar populations.

## 2. Materials and Methods

### 2.1. Study Design and Participants

This was a descriptive, cross-sectional, web-based study of QU students at all levels, faculty members, and administrative staff aged 18 years and above and living in Qatar. A link to the online survey was sent through QU email announcements. An online English and Arabic questionnaire was created using the Experience Management Platform Blue, which is an electronic data capture tool hosted at QU. The Blue system sends a unique link to each participant’s email to confirm that each participation is unique and that there are no duplicates. The questionnaire was distributed over a three-week period in February 2021. Data were collected anonymously, and the confidentiality of information was guaranteed. The QU Institutional Review Board granted ethical approval (study QU-IRB 1404-E/20). Informed consent was obtained from all participants after being given detailed information about the study but before they were given access to the questionnaire.

### 2.2. Instrument

The survey was modified from a previously validated and published questionnaire [11]. A face-to-face pilot test was conducted in ten participants to assess questionnaire clarity in Arabic and English. The survey instrument consisted of 48 questions in 8 sections (survey questions in English and Arabic are provided in Supplementary Materials Table S1). The first part of the survey contained six questions on demographics including gender, age, nationality, student or employee, college, and level of education. Acceptance of a COVID-19 vaccine was assessed by the statement “Once a COVID-19 vaccine is made publicly available…”, then the participant could choose either “I would get vaccinated” or “I would not get vaccinated”. Participants’ knowledge about the disease, its symptoms, preventative methods, transmission, and treatment were evaluated by asking true (score 1) and false (score 0) questions, and the sum of correct answers was used as a knowledge score (range 0–7 points). The survey measured multiple factors that we hypothesized might contribute to COVID-19 vaccine attitudes and acceptance including length of vaccine development, vaccine efficacy (50%, 75%, or 99% vaccine effectiveness), vaccine side effects, previous exposure of the participant, a family member, friend, or colleague to COVID-19, and attitude toward similar vaccines.

### 2.3. Statistical Analysis and Sample Size Calculation

Descriptive statistics and cross-tabulation (with chi-squared and *p*-values) were used to test for significant differences in the distribution of participant demographics and willingness to receive a COVID-19 vaccine. Chi-squared tests were also used to evaluate all categorical variables including factors that might influence willingness to take the vaccine. Factors affecting willingness to take the vaccine were also analyzed by multinomial logistic regression, with odds ratios (OR), 95% confidence intervals (95% CI) and *p*-values ≤ 0.05 calculated for each independent variable. This test adjusted for age, sex, educational level, and nationality (Qatari or non-Qatari). All analyses were conducted using SPSS version 26 (IBM Corporation, New York, NY, USA), and a *p*-value less than 0.05 was considered statistically significant. The sample size was calculated using the Survey System (https://www.surveysystem.com/sscalc.htm, accessed on 5 May 2021), which established that, for a 95% CI and 5% margin of error, the minimum sample size needed should be 361.

## 3. Results

### 3.1. Sociodemographic Characteristics

A total of 462 responses were collected from QU employees and students. The sociodemographic characteristics of the participants are summarized in Table 1. Half of participants were employees and the other half were students (n = 231 employees and n = 231 students). The majority of respondents were female (n = 289, 62.6%), and slightly more non-Qatari nationals responded to the survey (n = 254, 55%) than non-nationals. In the sample, 58.4% of respondents held a diploma (a two-year college degree) or were registered for an undergraduate degree, while 41.6% held or were registered for a postgraduate degree (MSc/PhD). Employees and students from different colleges participated in the survey.

### 3.2. Vaccine Acceptance

**Figure 1:**
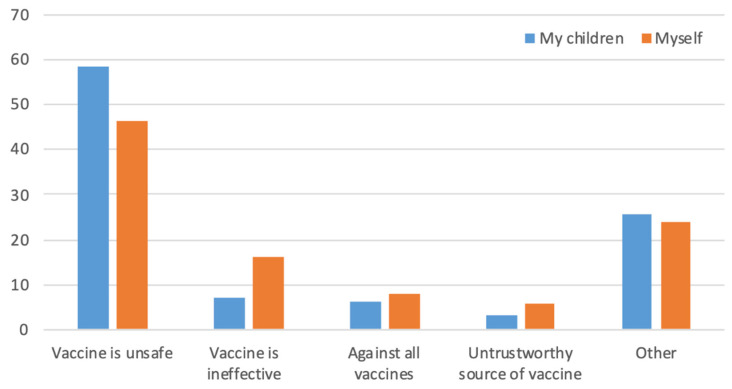
Reasons given for unwillingness to take the COVID-19 vaccine in 170 participants or their children. The y-axis represents % responses. Concern about vaccine safety was a major reason for vaccine hesitancy.

A total of 454 out of 462 (response rate 98.3%) participants responded to the question about willingness to take the vaccine. Of these, 62.6% (284/454) of participants were agreeable to COVID-19 vaccination, while 37.4% (170/454) responded with “I would not be vaccinated”. When we asked the same question regarding vaccinating their children, 200/462 individuals indicated that they had children and 92 (46%) of these said they would not vaccinate their children. Respondents who answered that they were unwilling to take the vaccine or to give it to their children were asked for their reasons. Safety and effectiveness were the main concerns regarding self-vaccination (46% and 16%, respectively), while safety was the main reason given for not vaccinating their children (58%). Respectively, 8% and 6% of participants did not believe in giving any vaccine to themselves or their children (Figure 1).

A higher rate of COVID-19 vaccine acceptance was seen in males than females (*p* ≤ 0.001). In addition, vaccine acceptance increased with age, with the highest acceptance seen in the 45+ years age group (*p* ≤ 0.001). In addition, participants from the colleges of health-related sciences (including the College of Medicine, College of Pharmacy, College of Health Sciences, and College of Dental Medicine) showed a higher rate of vaccine acceptance. Non-Qataris, QU employees, and higher educational level groups were also associated with higher rates of COVID-19 vaccine acceptance (*p* ≤ 0.001) (Table 2).

### 3.3. Knowledge Levels about COVID-19 Disease and Vaccines

Of the participants, 53% had high, 37% medium, and 10% low knowledge scores. There was no significant difference between the three groups in terms of willingness to vaccinate against COVID-19 (Table 3; chi-squared test). Sub-analysis revealed no significant differences according to age and gender (Table S2).

### 3.4. Attitudes and Beliefs towards COVID-19 Disease and Vaccine

**Figure 2:**
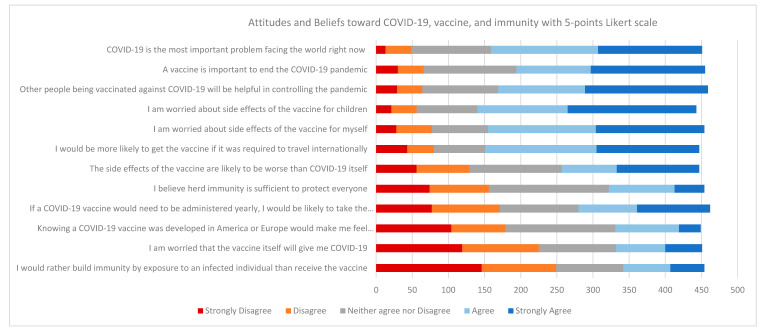
Attitudes and beliefs toward COVID-19 disease, vaccination, and immunity using a five-point Likert scale.

The attitudes and beliefs of QU employees and students regarding COVID-19 disease, vaccination, and immunity using a Likert five-point scale are shown in Figure 2. Most participants agreed that the COVID-19 pandemic was serious, with 65% answering “agree” and “strongly agree” to the statement “COVID-19 is the most important problem facing the world right now”. Moreover, 63% agreed or strongly agreed with the statement “Other people being vaccinated against COVID-19 will be helpful in controlling the pandemic”. Participants showed a high degree of concern regarding side effects of the COVID-19 vaccine to themselves or their children, with 68% answering “agree” or “strongly agree” to the statement “I am worried about the side effects of the vaccine for my children” and 66% answering the same regarding self-vaccination. Participants were evenly split regarding beliefs about herd immunity being sufficient to protect everyone, with 29% agreeing or strongly agreeing, 37% neither agreeing nor disagreeing, and 34% disagreeing or strongly disagreeing. When asked about taking the vaccine as a condition for international travel, 66% agreed or strongly agreed and 28% disagreed or strongly disagreed. Considering the location of vaccine development, 40% of participants disagreed or strongly disagreed with the statement “Knowing a COVID-19 vaccine was developed in America or Europe would make me feel more comfortable”, 34% neither agreed nor disagreed, and 27% agreed or strongly agreed. Finally, 24% responded with “agree” or “strongly agree” to the statement “I would rather build immunity by exposure to an infected individual than receive the vaccine”.

### 3.5. Other Determinants Influencing COVID-19 Vaccine Hesitancy and Acceptance

**Figure 3:**
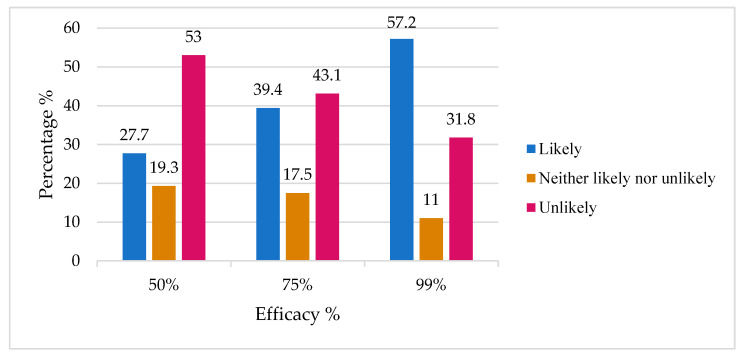
Different levels of vaccine efficacy and participants’ responses on how likely they were to be vaccinated.

Table 4 shows the relationship between some predictive factors influencing the decision to accept the vaccine. Participants who considered it important to take the annual influenza vaccination were less hesitant to accept the COVID-19 vaccine (*p* < 0.001). Participants who reported that it is important or somewhat important to get the flu vaccine every year showed 8.51 and 3.38 times increase, respectively, in accepting to be vaccinated against COVID-19. However, among participants who indicated that it is important to take the influenza vaccine, 13% indicated that they would not take the COVID-19 vaccine. With regard to the source of news and information on COVID-19, 215 out of 448 (48%) respondents said that they relied in professional sources of information (primary doctor, local health authority, or WHO), but the source of information was not associated with a willingness or unwillingness to be vaccinated. In addition, the source of information was not associated with the knowledge score (data not shown). We examined the effect of previous COVID-19 exposure or knowing someone infected with COVID-19 (including family members, friends, or coworkers) on willingness to take the vaccine. Previous exposure and the severity of the experience were not associated with a willingness or unwillingness to be vaccinated. There was a lower willingness (OR: 0.26, CI (0.12–0.54)) to be vaccinated against COVID-19 due to participants’ worries about the rushed pace of development of a COVID-19 vaccine and the failure to detect potential side effects. In addition, 42% (190/454) indicated that the minimum length of testing should be over three years for them to feel comfortable with the vaccine. We asked participants about their willingness to take the vaccine with three different levels of hypothetical efficacy (50%, 75%, or 99%). As the percentage efficacy increased, respondents reported that they were more likely to be vaccinated (Figure 3).

## 4. Discussion

By 28 March 2021, the official record from the Ministry of Public Health (MOPH) in Qatar showed that ~22% of adults in Qatar had received at least the first dose of a COVID-19 vaccine (https://covid19.moph.gov.qa, accessed on 3 April 2021). The vaccination campaign started on 23 December 2020 after MOPH approval of the Pfizer and BioNTech and Moderna vaccines. We conducted our survey of a representative education sector population in February 2021, after the vaccination program had started in Qatar. Although most surveyed QU students and employees recognized the severity of the COVID-19 pandemic and that vaccination is the best solution for social recovery, less than 63% of our cohort accepted being vaccinated. This figure is less than the threshold for a population to gain herd immunity. The threshold for COVID-19 herd immunity is unknown, but most studies suggest that it should be more than 75% if the vaccine efficacy is less than 100% [2]. Vaccine hesitancy is an important factor in gaining herd immunity in a society; however, it is not the only factor. Other factors such as the emergence of new variants and the delayed arrival of child vaccination also play important roles in developing herd immunity [12].

In the UK, a study by University College London’s Virus Watch showed that the UK acceptance rate increased after the vaccination program had started, with 86% of participant who rejected the vaccine in December 2020 going on to accept taking the vaccine in February 2021 [13]. A general Qatari population survey conducted by Alabdulla et al. from mid-October to mid-November 2020, before the vaccination program started, showed that almost 61% of participants said they would “definitely” or “probably” accept the vaccine [14]. Although the survey structure was different to ours and we targeted a specific sector of the Qatari population, the acceptance rates between these two studies are very similar. This suggests that, even after the start of the vaccination campaign in Qatar, vaccine hesitancy persists, as shown by Alabdulla et al. [14] and our data. Similar results have been reported in other Gulf countries. For example, a study from Kuwait showed that vaccine acceptance dropped from 73% to 47% among Kuwaiti individuals during the pandemic [15]. In Jordan, there was 36.8% hesitancy towards the COVID-19 vaccine [16], and another study of healthcare workers in Arab countries reported a similar hesitancy range of 25.8% to 32.8% [17]. A decline in vaccine acceptance has also been reported in a few studies. For example, an Australian study showed a decline, although not significant, in vaccine acceptance during the early stages of the pandemic [18]. The UK experience of increased vaccine acceptance after the vaccination program started may represent a special case: a study conducted during the early stages of the pandemic showed that the UK population has a relatively high vaccine acceptance rate among European populations [19]. In Qatar, the government has started several initiatives to boost COVID-19 vaccine acceptance (https://covid19.moph.gov.qa, accessed on 3 April 2021); however, our survey showed that hesitancy is still high, at least in this population.

Similar to other populations worldwide, the demographic analyses of our cohort showed that men are more likely to accept the vaccine and that women are more cautious and anxious about vaccination [3]. Although the overall vaccine acceptance was 63%, almost 80% of participants with or taking a postgraduate degree (MScs and PhDs) were willing to take the vaccine. Approximately 50% of our participants indicated that they had children, but 46% of them said that they were unwilling to vaccinate their children, with safety cited as the main reason for this decision. The idea that vaccines have a negative impact on the future health of a child persists, despite an abundance of research showing the opposite [20]. We found that 90% of participants had a moderate to high knowledge score, but knowledge was not associated with intent to vaccinate. About half of our students had no intention to be vaccinated (115 out of 231, 50.2%). A study of Italian university students reported 86% vaccine acceptance among 735 students [21]. This finding highlights an important group of the Qatari population that needs special attention. Although the number of participants from colleges of health-related sciences (including the College of Medicine, College of Pharmacy, College of Health Sciences, and College of Dental Medicine) was small (only 36 participants), this group showed a higher rate of vaccine acceptance compared to other non-health related disciplines, consistent with international studies where healthcare workers have higher acceptance rates of vaccination [22].

Approximately half (45%) of participants indicated that it is “important” or “somewhat important” for them to take the influenza vaccine every year. Despite the differences between COVID-19 and influenza vaccines, for example an efficacy of almost 95% for the COVID-19 vaccine and 40–60% for the flu vaccine, there was high acceptance of the COVID-19 vaccine among participants who accepted the influenza vaccine in our cohort. This is consistent with other studies in which a history of influenza vaccination was associated with an increased acceptance rate for the COVID-19 vaccine [23,24]. One explanation is that even though these diseases are different, they share similar features and symptoms. Interestingly, among participants who indicated that it is important to take the influenza vaccine, 13% indicated that they would not take the COVID-19 vaccine, which may be due to uncertainty or lack of trust in the new vaccine.

The primary source of COVID-19 news was not associated with the intent to vaccinate nor with the knowledge score. This is in contrast with some other studies, where people receiving COVID-19 information from social media had higher levels of vaccine hesitancy [25]. Our participants were neutral to the geographic origin of vaccine development, as they did not favor a vaccine developed in America or Europe over any other country. Finally, in an open-ended question, we asked our participants “What would make you most comfortable with the idea of receiving a vaccine for COVID-19?”. The majority of respondents mentioned that knowing all the side effects of the vaccine was the most important factor. Continually hearing about severe side effects from vaccinated individuals made others more hesitant towards the vaccine, highlighting the need for vaccine communications teams to proactively spread successful cases and statistics [26].

A major limitation of our study is the time of survey release. We sent the email announcement less than two months after the vaccination program had started in Qatar. This could be a major limitation, as people may change their attitudes with time. Another concern is the spread of the new UK variant in Qatar during January and February 2021, which may have contributed to hesitancy in our population. A repetition of the same study would be useful to monitor people’s attitudes over time. Finally, this was a web-based survey, which might be associated with self-selection and other biases such as social desirability, where participants answer the questions to meet the researcher’s expectation or following “common sense” rather than actual knowledge [27].

## 5. Conclusions

In this representative education sector cohort, vaccine hesitancy was similar to that seen at the global level. Our students were more hesitant than reported for students from other universities worldwide. This hesitancy could hamper achieving herd immunity. Interestingly, news from lay sources such as social media had no influence in accepting or rejecting the vaccine in our cohort. Vaccine safety and efficacy are two major factors affecting willingness to accept the vaccine in our cohort. However, people’s attitudes are likely to change as more data and information about the COVID-19 vaccine become available, so continuous research to track factors influencing vaccine acceptance will help policymakers to take suitable measures in near real-time.

## Figures and Tables

**Table 1:** Sociodemographic variables of the study population (n = 462).

Demographic	Variable Category	n	%
Gender	Male	173	37.4
	Female	289	62.6
Age	18–24 years	151	32.7
	25–34 years	117	25.3
	35–44 years	85	18.4
	45+ years	109	23.6
Nationality	Qatari	208	45
	Non-Qatari	254	55
University Status	Student	231	50
	Employee	231	50
Colleges	Arts and Sciences	107	23.2
	Business and Economics	76	16.5
	Engineering	66	14.3
	Education	40	8.7
	Law	31	6.7
	Health Sciences	20	4.3
	Islamic Studies	17	3.7
	Medicine	9	1.2
	Pharmacy	6	1.3
	Dental Medicine	1	0.2
	Others (including general services and presidency)	82	17.7
Education Level	Diploma/undergraduate	270	58.4
	Postgraduate	192	41.6

**Table 2:** Participant characteristics by COVID-19 vaccine acceptance.

Demographic	Variable Category	Would Vaccinate n (%)	Would Not Vaccinate n (%)	X^2^	*p*-Value
Gender Age	Male Female 18–24 years 25–34 years 35–44 years 45+ years	123 (72.9) 161 (56.7) 77 (51) 61 (53.5) 61 (73.5) 85 (80.2)	46 (27.1) 124 (43.3) 74 (49) 53 (46.5) 22 (26.5) 21 (19.8)	12.02 30.91	<0.001 <0.001
Nationality QU Status	Qatari Non-Qatari Student Employee	105 (51.2) 179 (71.9) 114 (49.8) 170 (75.6)	100 (48.8) 70 (28.1) 115 (50.2) 55 (24.4)	20.50 32.19	<0.001 <0.001
Education level	Diplomas/Bachelors Masters/PhDs	136 (50.9) 148 (79.1)	131 (49.1) 39 (20.9)	37.39	<0.001
Health-related discipline	Health colleges	29 (80.6)	7 (19.4)		
	Non-health colleges	255 (61)	163 (39)	5.40	0.02

**Table 3:** Relationship between COVID-19 knowledge score and willingness to vaccinate.

Knowledge Score	Would Vaccinate n (%)	Would Not Vaccinate n (%)	Statistic	*p*-Value
Low (0–3)	30 (73.2)	11 (26.8)	X2 = 2.6	0.27
Medium (4–5)	100 (59.5)	68 (40.5)		
High (6–7)	154 (62.9)	91 (37.1)		

**Table 4:** Relationship between factors and determinants influencing acceptance and willingness to vaccinate.

Question	Answers	Would Vaccinate n (%)	Would Not Vaccinate n (%)	Multinomial Logistic Regression Analysis		
				Odd ratio *	95% CI	*p*
How important is it for you to get the flu vaccine every year?	Important	93 (86.9)	14 (13.1)	8.51	4.37–16.5	<0.0001
	Somewhat important	75 (75.8)	24 (24.2)	3.38	1.94–5.88	<0.0001
	Not important	116 (46.8)	132 (53.2)	Reference		
What is your primary source of information regarding COVID-19?	Professional source	139 (65.3)	74 (34.7)	2.39	0.28–20.1	0.43
	Unprofessional source	141 (60)	94 (40)	1.81	0.21–15.5	0.59
	Leaders	2 (50)	2 (50)	Reference		
How severe were the symptoms of COVID-19 infection for yourself?	Severe	1 (16.7)	5 (9.5)	0.21	0.02–2.01	0.17
	Moderate	16 (57.1)	12 (42.9)	1.12	0.44–2.9	0.79
	Not at all	54 (58.7)	38 (41.3)	Reference		
How severe were the symptoms of COVID-19 infection for your family member?	Severe	21 (51.2)	20 (48.8)	0.75	0.33–1.7	0.49
	Moderate	71 (62.3)	43 (37.7)	1.21	0.65–2.3	0.53
	Not at all	50 (58.8)	35 (41.2)	Reference		
How severe were the symptoms of COVID-19 infection for your friend?	Severe	20 (69%)	9 (31)	1.39	0.54–3.61	0.49
	Moderate	77 (57.5)	57 (42.5)	0.92	0.52–1.62	0.77
	Not at all	57 (58.8)	40 (41.2)	Reference		
How severe were the symptoms of COVID-19 infection for coworker?	Severe	6 (50)	6 50)	0.10	0.08–1.26	0.10
	Moderate	54 (62.8)	32 (37.2)	0.57	0.29–1.16	0.12
	Not at all	64 (66)	33 (34)	Reference		
What is the minimum length of time a testing process would take to make you feel comfortable with COVID-19 vaccine?	3 months–1 year	139 (89.1)	17 (10.9)	13.1	7.01–24.0	<0.0001
	1–2 years	68 (63)	40 (37)	2.91	1.71–4.91	<0.0001
	3+ years	77 (40.5)	113 (59.5)	Reference		
I worry that the rushed pace of testing for a COVID-19 vaccine will fail to detect potential side effects.	Agree	155 (52.9)	138 (47.1)	0.26	0.12–0.54	<0.0001
	Neither agree nor disagree	80 (78.4)	22 (21.6)	0.75	0.32–1.76	0.50
	Disagree	49 (83.1)	10 (16.9)	Reference		

* Odds ratio was adjusted for age, sex, educational level, and nationality (Qatari or non-Qatari).

## Data Availability

Not applicable.

## Supplementary Materials

The following are available online at https://www.mdpi.com/article/10.3390/vaccines9060665/s1, Table S1: survey questions in English and Arabic, Table S2: Subanalysis of the knowledge score by age and gender.
