# Supplementary material for: COVID-19 Vaccine Hesitancy in a Representative Education Sector Population in Qatar
Source: Vaccines (Basel). 2021 Jun 18;9(6):665. doi: 10.3390/vaccines9060665 (PMC8235273; doi:10.3390/vaccines9060665)
Supplement: Supplementary file 1 [file vaccines-09-00665-s001.zip › vaccines-1195768-supplementary.pdf]

COVID-19 vaccine questionnaire

1.1 Gender

- ○ Male
- ○ Female

1.2 Age

- ○ 18 – 24 years
- ○ 25 – 34 years
- ○ 35 – 44 years
- ○ 45 or older

1.3 Nationality

- ○ Qatari
- ○ Non-Qatari

1.4 Are you a

- ○ Student
- ○ Faculty member
- ○ Administrated staff

1.5 College

- ○ Business and Economics
- ○ Arts and Sciences
- ○ Education
- ○ Engineering
- ○ Health Sciences
- ○ Law

- ○ Medicine
- ○ Sharia and Islamic Studies
- ○ Pharmacy
- ○ Dental Medicine
- ○ Other (Specify)

1.6 Current level of education

- ○ Diploma
- ○ Undergraduate
- ○ Masters
- ○ PhD
- ○ Other (Specify)

2.1 Please select ALL of the following statements that apply to your experience with COVID-19:

- ☐ I have tested positive for a COVID-19 infection
- ☐ A family member has tested positive for COVID-19 infection
- ☐ A friend has tested positive for COVID-19 infection
- ☐ A coworker has tested positive for COVID-19 infection
- ☐ I do not know anyone who has tested positive for COVID-19 infection

Display This Question:

If Q2.1 = I have tested positive for a COVID-19 infection

2.2 How severe was *your COVID-19 infection*?

- ○ No symptoms/mild symptoms
- ○ Moderate symptoms but health care providers were not contacted
- ○ Moderate symptoms and health care providers were contacted
- ○ Severe symptoms/hospitalization

Display This Question:

If Q2.1 = A family member has tested positive for COVID-19 infection

2.3 How severe were the symptoms of the COVID-19 infection of *your family member?*

- ○ No symptoms/mild symptoms
- ○ Moderate symptoms but health care providers were not contacted
- ○ Moderate symptoms and health care providers were contacted
- ○ Severe symptoms/hospitalization
- ○ Death

Display This Question:

If Q2.1 = A friend has tested positive for COVID-19 infection

2.4 How severe were the symptoms of the COVID-19 infection of your friend?

- ○ No symptoms/mild symptoms
- ○ Moderate symptoms but health care providers were not contacted
- ○ Moderate symptoms and health care providers were contacted
- ○ Severe symptoms/hospitalization
- ○ Death

Display This Question:

If Q2.1 = A coworker has tested positive for COVID-19 infection

2.5 How severe were the symptoms of the COVID-19 infection of your coworker?

- ○ No symptoms/mild symptoms
- ○ Moderate symptoms but health care providers were not contacted
- ○ Moderate symptoms and health care providers were contacted
- ○ Severe symptoms/hospitalization
- ○ Death

2.6 How has your experience with the COVID-19 global pandemic affected your opinion on vaccinations in general?

- ○ I am much more likely to vaccinate myself/my children
- ○ I am more likely to vaccinate myself/my children
- ○ My opinion on vaccinations has not changed
- ○ I am less likely to vaccinate myself/my children
- ○ I am much less likely to vaccinate myself/my children

2.7 How has the COVID-19 pandemic affected your mental health?

- ○ Much better
- ○ Somewhat better
- ○ About the same
- ○ Somewhat worse
- ○ Much worse

2.8 How has the COVID-19 pandemic affected your ability to carry out your normal activities?

- ○ Dramatic restrictions
- ○ Moderate restrictions
- ○ No restrictions
- ○ Moderately easier
- ○ Much easier

3.1 How closely do you follow news regarding COVID-19?

- ○ Very closely
- ○ Somewhat closely
- ○ An average amount
- ○ Not very closely
- ○ Not at all

3.2 What is your primary source of information regarding COVID-19?

- ○ Your primary doctor
- ○ Local Health Authority or WHO (World Health Organization)
- ○ Local news
- ○ Friends or Social Media
- ○ Celebrities/public figures
- ○ Religious leaders
- ○ Political leaders
- ○ Other (Please specify)

4.1 Vaccines against pneumonia can protect against COVID-19

- ○ True 0
- ○ False 1

4.2 Certain antibiotics can prevent and/or treat COVID-19.

- ○ True 0
- ○ False 1

4.3 On average it takes 5–6 days from when someone is infected with COVID-19 for symptoms to show, however it can take up to 14 days.

- ○ True 1
- ○ False 0

4.4 Regularly rinsing your nose with saline can help prevent infection with COVID-19.

- ○ True 0
- ○ False 1

4.5 Once you contract COVID-19, the virus can never be eliminated from your body.

- ○ True 0
- ○ False 1

4.6 Symptoms of COVID-19 can include sore throat, diarrhea, and conjunctivitis (eye infection).

- ○ True 1
- ○ False 0

4.7 Most people who contract COVID-19 will recover from it.

- ○ True 1
- ○ False 0

5.1 I take all the vaccines recommended by my primary care physician.

- ○ I take them all
- ○ I take most
- ○ I take some
- ○ I donot take any
- ○ I am uncertain

5.2 How important is it for you to get the flu vaccine every year?

- ○ Very important
- ○ Important
- ○ Somewhat important
- ○ Not very important
- ○ Not at all important

Please indicate how you would respond in the following scenarios.

5.3 Once a COVID-19 vaccine is made available

○ I would vaccinate myself

○ I would not vaccinate myself

Display This Question:

If Q5.3 = I would not vaccinate myself

5.4 Which of these statements most closely resembles your reason for choosing to not vaccinate yourself:

○ I do not believe the vaccine is safe

○ I do not believe the vaccine is effective

○ I do not trust the source that encouraged me to get the vaccine

○ I do not believe in any vaccines, and my reason is not any different for a new COVID-19 vaccine

○ A source that I trust encouraged me to NOT get the vaccine

○ I am indifferent to receiving the vaccine, but will probably end up not receiving it

○ Other

5.5 Once a COVID-19 vaccine is made available

○ I do have children and I would vaccinate them

○ I do have children and I would not vaccinate them

○ I do not have children

Display This Question:

If Q5.5 = I do have children and I would not vaccinate them

5.6 Which of these statements most closely resembles your reason for choosing to not vaccinate

your children:

- I do not believe the vaccine is safe for children
- I do not believe the vaccine is effective
- I do not trust the source that encouraged me to give my child the vaccine
- I do not believe in any vaccines, and my reason is not any different for a new COVID-19 vaccine
- A source that I trust encouraged me to NOT give my child the vaccine
- I am indifferent to having my child receive the vaccine, but probably will not end up having my child receive it
- Other

6.1 Will you be willing to vaccinate with the COVID-19 vaccine if it was recommended by your employer?

- I would vaccinate myself
- I would not vaccinate myself

6.2 Do your religious or cultural beliefs go against vaccinations (e.g non-Halal source in vaccine)?

- Yes
- No

6.3 If a COVID-19 vaccine would need to be administered yearly (similar to the flu shot), how likely would you be vaccinated?

- Extremely likely (almost every year)
- Very likely
- Somewhat likely
- Not very likely
- Not at all likely (almost never)

Display This Question:

If Q6.3 = Not very likely

And Q6.3 = Not at all likely (almost never)

6.4 Which of the following most closely describes why you would be unlikely to receive a yearly vaccination for yourself?

- ○ Limited time
- ○ Limited access to health care
- ○ Concern with the vaccine itself
- ○ Other

6.5 If a vaccine for COVID-19 would protect 50% of the people who received it, how likely would you be vaccinated?

- ○ Extremely likely
- ○ Somewhat likely
- ○ Neither likely nor unlikely
- ○ Somewhat unlikely
- ○ Extremely unlikely

6.6 If a vaccine for COVID-19 would protect 75% of those who received it, how likely would you be vaccinated?

- ○ Extremely likely
- ○ Somewhat likely
- ○ Neither likely nor unlikely
- ○ Somewhat unlikely
- ○ Extremely unlikely

6.7 If a vaccine for COVID-19 would protect 99% of those who received it, how likely would you be vaccinated?

- ○ Extremely likely
- ○ Somewhat likely
- ○ Neither likely nor unlikely
- ○ Somewhat unlikely
- ○ Extremely unlikely

6.8 Other people being vaccinated against COVID-19 will be helpful in controlling the pandemic

- ○ Strongly agree
- ○ Somewhat agree
- ○ Neither agree nor disagree
- ○ Somewhat disagree
- ○ Strongly disagree

7.1 Vaccines are important for the prevention of serious diseases.

- ○ Strongly agree
- ○ Agree
- ○ Uncertain
- ○ Disagree
- ○ Strongly disagree

7.2 The administration of more than one vaccine at the same time can be unsafe for my child's health.

- ○ Strongly agree
- ○ Agree
- ○ Uncertain
- ○ Disagree
- ○ Strongly disagree

7.3 The side effects of most vaccines outweigh the benefits

- ○ Strongly agree
- ○ Agree
- ○ Uncertain
- ○ Disagree
- ○ Strongly disagree

7.4 I worry that the rushed pace of testing for a new COVID-19 vaccine will fail to detect potential side effects or dangers.

- ○ Strongly agree
- ○ Agree
- ○ Uncertain
- ○ Disagree
- ○ Strongly disagree

7.5 A vaccine is important to end the COVID-19 pandemic

- ○ Strongly agree
- ○ Somewhat agree
- ○ Neither agree nor disagree
- ○ Somewhat disagree
- ○ Strongly disagree

8.1 What is the minimum length of time a testing process would take that would make you feel comfortable with a COVID-19 vaccine?

- ○ 3-6 months
- ○ 6 months to a year
- ○ 1-2 years
- ○ Between 2 and 5 years
- ○ More than 5 years

8.2 Please rank from 1-5 how much you agree with the following statements, where 1 is Strongly Disagree and 5 is Strongly Agree

	1 (Strongly Disagree)	2 (Disagree)	3 (Neither agree nor Disagree)	4 (Agree)	5 (Strongly Agree)
I am worried that the vaccine itself will give me COVID-19.	○	○	○	○	○
I would rather build immunity by exposure to an infected individual than receive the vaccine.	○	○	○	○	○
I would be more likely to get the vaccine if it was required to travel internationally.	○	○	○	○	○
I believe herd immunity is sufficient to protect everyone.	○	○	○	○	○
I am worried about the cost of a COVID-19 vaccine	○	○	○	○	○

I am worried about side effects of the vaccine for myself.	○	○	○	○	○
I am worried about side effects of the vaccine for children.	○	○	○	○	○
The side effects of the vaccine are likely to be worse than COVID-19 itself	○	○	○	○	○
Knowing a COVID-19 vaccine was developed in America or Europe would make me feel more comfortable receiving it	○	○	○	○	○
Knowing a COVID vaccine was developed somewhere other than America or Europe would make me feel more comfortable receiving it	○	○	○	○	○

8.3 How much of a problem is COVID-19 in Qatar and to the world?

- Not a problem at all
- Insignificant compared to other problems
- Somewhat of a problem
- A severe problem, more important than most other issues
- The most important problem facing the world right now

8.4 Which vaccine do you think more safe?

- Oxford-AstraZeneca vaccine produced in the UK
- Pfizer vaccine produced in the USA
- No difference

8.5 Please answer the following questions in your own words: The biggest fear I have about a COVID-19 vaccine is...

<type here>

8.6 Please answer the following question in your own words: What would make you the most comfortable with the idea of receiving a vaccine for COVID-19?

<type here>

ما مدى استعدادك لأخذ مطعوم كوفيد19 (COVID-19) ؟

عزيزي المُشارك المعلومات الواردة في هذا الاستبيان ما هي إلا لغرض البحث والتعليم فقط، حيث أن هوية المُشارك مجهولة وسيتم التعامل مع الإجابات بشكلٍ سري، للمشاركة في هذه الدراسة يجب عليك أن تكون طالبًا أو عضوًا في هيئة التدريس أو موظفًا في جامعة قطر، كما أن عمر المشارك ١٨ عامًا فما فوق، يرجى الإجابة بصدق واختيار الحقل المناسب. شكرًا لتعاونكم.

1.1الجنس
- ذكر
- أنثى

1.2 العمر
- 18 - 24
- 25 - 34
- 35 - 44
- 45 أو أكبر

1.3 الجنسية
- قطري
- غير قطري

1.4 هل أنت
- طالب
- عضو هيئة تدريس
- الموظفين الإداريين

1.5 الكلية
- الأعمال والاقتصاد
- الآداب والعلوم
- التعليم
- الهندسة
- العلوم الصحية
- القانون
- الطب
- الشريعة والدراسات الإسلامية
- الصيدلة
- طب الأسنان
- أخرى (يرجى التحديد)

1.6 المستوى التعليمي الحالي
- دبلوم
- المرحلة الجامعية
- الماجستير
- دكتوراه
- أخرى (يرجى التحديد

2.1 يرجى تحديد جميع العبارات التالية التي تنطبق على تجربتك مع COVID- 19

- لقد ثبتت إصابتي بعدوى COVID-19
- ثبتت إصابة أحد أفراد الأسرة بعدوى COVID-19
- ثبتت إصابة صديق بعدوى COVID-19
- ثبتت إصابة زميل في العمل بعدوى COVID-19
- لا أعرف أي شخص ثبتت إصابته بعدوى COVID-19

اعرض هذا السؤال:
إذا كانQ2.1 = لقد ثبتت إصابتي بعدوى COVID-19

2.2 ما مدى شدة اصابتك بـ COVID-19؟

- لا توجد أعراض / أعراض خفيفة
- أعراض معتدلة ولكن لم يتم الاتصال بمقدمي الرعاية الصحية
- أعراض معتدلة وتم الاتصال بمقدمي الرعاية الصحية
- أعراض شديدة / دخول المستشفى

اعرض هذا السؤال:
إذا كانQ2.1 = لقد ثبتت إصابة احد افراد الاسرة بعدوى COVID-19

2.3 ما مدى شدة أعراض عدوى COVID-19 لأفراد عائلتك ؟

- لا توجد أعراض / أعراض خفيفة
- أعراض معتدلة ولكن لم يتم الاتصال بمقدمي الرعاية الصحية
- أعراض معتدلة وتم الاتصال بمقدمي الرعاية الصحية
- أعراض شديدة / دخول المستشفى
- الموت

اعرض هذا السؤال:
إذا كانQ2.1 = لقد ثبتت إصابة صديق بعدوى COVID-19

2.4 ما مدى شدة أعراض عدوى COVID-19 لصديقك ؟

- لا توجد أعراض / أعراض خفيفة
- أعراض معتدلة ولكن لم يتم الاتصال بمقدمي الرعاية الصحية
- أعراض معتدلة وتم الاتصال بمقدمي الرعاية الصحية
- أعراض شديدة / دخول المستشفى
- الموت

اعرض هذا السؤال:
إذا كانQ2.1 = ثبتت إصابة زميل في العمل بعدوى COVID-19

2.5 ما مدى شدة أعراض عدوى COVID-19 لزميلك في العمل؟

- لا توجد أعراض / أعراض خفيفة
- أعراض معتدلة ولكن لم يتم الاتصال بمقدمي الرعاية الصحية
- أعراض معتدلة وتم الاتصال بمقدمي الرعاية الصحية
- أعراض شديدة / دخول المستشفى
- الموت
- أعراض شديدة / دخول المستشفى
- الموت

2.6 كيف أثرت تجربتك مع جائحة COVID-19 العالمي على رأيك في التطعيمات بشكل عام؟

- انا اكثر احتمالا أن أقوم بتطعيم نفسي / أطفالي

- احتمال أن أقوم بتطعيم نفسـي / أطفالي
- رأيي في التطعيمات لم يتغير
- أنا أقل احتمالا لتطعيم نفسـي / أطفالي
- أنا أقل احتمالا بكثير لتطعيم نفسـي / أطفالي

2.7 كيف اثر جائحة COVID-19 على صحتك العقلية؟
- أفضل بكثير
- أفضل الي حد ما
- عن نفسه
- أسوأ الى حد ما
- أسوأ بكثير

2.8 كيف اثر جائحة COVID-19 على قدرتك على القيام بأنشطتك المعتادة؟
- قيود كبيرة
- قيود معتدلة
- لا قيود
- أسهل الى حد ما
- أسهل بكثير

3.1 إلى أي مدى تتابع عن الأخبار المتعلقة في COVID-19؟
- اتابع بكثره
- اتابع إلى حد ما
- اتابع باعتدال
- لا اتابع كثيرا
- لا اتابع على الأطلاق

3.2 ما هو مصدرك الأساسي للمعلومات المتعلقة ب COVID-19؟
- طبيبك الاساسي
- هيئة الصحة المحلية أو منظمة الصحة العالمية (منظمة الصحة العالمية)
- الأخبار المحلية
- الأصدقاء أو وسائل التواصل الاجتماعي
- مشاهير / شخصيات عامة
- القادة الدينيون
- القادة السياسيون
- أخرى (يرجى التحديد)

4.1 يمكن للقاحات ضد الالتهاب الرئوي أن تحمي منCOVID-19
- صحيح
- خطأ

4.2 يمكن لبعض المضادات الحيوية أن تمنع و / أو تعالج.COVID-19
- صحيح
- خطأ

4.3 في المتوسط ، يستغرق الأمر من 5 إلى 6 أيام من إصابة شخص ما بفيروس COVID-19 حتى تظهر الأعراض ، ومع ذلك قد يستغرق الأمر ما يصل إلى 14 يومًا.
- صحيح
- خطأ

4.4 شطف الأنف بانتظام بمحلول ملحي يمكن أن يساعد في منع الإصابة بـ.COVID-19
- صحيح
- خطأ

4.5 بمجرد إصابتك بـCOVID-19 ، لا يمكن القضاء على الفيروس من جسمك.
- صحيح
- خطأ

4.6 يمكن أن تشمل أعراض COVID-19 التهاب الحلق والإسهال والتهاب الملتحمة (عدوى العين).
- صحيح
- خطأ

4.7 يتعافى معظم الأشخاص المصابين بـ COVID-19 منه.
- صحيح
- خطأ

5.1 أنا أتلقى التطعيمات التي أوصى بها طبيب الرعاية الأولية.

- أنا منتظم على كل المطاعيم
- أنا منتظم في معظم المطاعيم
- أنا منتظم على بعض المطاعيم
- أنا لست منتظم على أي من المطاعيم
- أنا غير متأكد

5.2 ما مدى أهمية الحصول على لقاح الإنفلونزا كل عام بالنسبة لك؟

- مهم جدا
- هام
- مهم إلى حد ما
- ليس مهم جدا
- ليس مهما على الإطلاق

5.3 يرجى توضيح كيف ستستجيب في السيناريوهات التالية إذا تم تطوير لقاح لمنع الإصابة بCOVID-19؟

إذا تم توفير اللقاح

- سأقوم بتلقيح نفسي
- لن أقوم بتلقيح نفسي

اعرض هذا السؤال:
اذا كان *5.3* = لن أقوم بتلقيح نفسي

5.4 أي من العبارات التالية يشبه إلى حد كبير سبب اختيارك عدم تلقيح نفسك:

- لا أعتقد أن اللقاح آمن
- لا أؤمن أن اللقاح فعال
- لا أثق بالمصدر الذي حثني على أخذ اللقاح
- لا أؤمن بأي لقاحات ، ولا يختلف سببي مطلقًا عن لقاح جديد لـCOVID-19
- مصدر أثق أ~~نه~~ به شجعني على عدم الحصول على اللقاح
- أنا غير مبال بتلقي اللقاح ، لكن من المحتمل أن ينتهي بي الأمر بعدم تلقيه
- أخرى

5.5 إذا تم توفير اللقاح

- لدي أطفال وسأقوم بتطعيمهم
- لدي أطفال ولن أقوم بتلقيحهم
- ليس لدي اطفال

اعرض هذا السؤال:
اذا كان *5.5* = لدي أطفال ولن أقوم بتلقيحهم

5.6 أي من العبارات التالية يشبه إلى حد كبير سبب اختيارك عدم تطعيم أطفالك:

- لا أعتقد أن اللقاح آمن للأطفال
- لا أؤمن أن اللقاح فعال
- لا أثق بالمصدر الذي شجعني على إعطاء طفلي اللقاح
- لا أؤمن بأي لقاحات ، وسببي لا يختلف عن لقاح جديد لـCOVID-19
- مصدر أثق به شجعني على عدم إعطاء اللقاح لطفل
- أنا غير مبال بتلقي طفلي اللقاح ، ولكن من المحتمل ألا ينتهي الأمر بتلقي طفلي
- أخرى

6.1 هل ستكون على استعداد للتطعيم بلقاح COVID-19 إذا أوصى به صاحب العمل؟

- سأقوم بتلقيح نفسي
- لن أقوم بتلقيح نفسي

6.2 هل ستظل تفكر في أخذ اللقاح إذا كانت معتقداتك الدينية أو الثقافية تتعارض مع اللقاحات (مثل المصدر غير الحلال في اللقاح) ؟

- نعم
- لا

6.3 إذا قدم لقاح COVID-19 متاحة للشعب العام، لكنه في حاجة إلى أن يؤخذ سنويا (على غرار لقاح الانفلونزا)، ما مدى احتمال أن تأخذ التلقيح؟

- مؤكد سوف اخذ اللقاح الى ابعد حد (تقريباً سنوياً)
- محتمل جدا
- محتمل الى حد ما
- غير محتمل كثيراً
- غير محتمل على الاطلاق

اعرض هذا السؤال:

اذا كان 6.3 = غير محتمل كثيراً
اذا كان 6.3 = غير محتمل على الاطلاق

6.4 أي مما يلي يصف بشكل وثيق لماذا من غير المحتمل أن تتلقى تطعيمًا سنويًا لنفسك؟

- محدود الوقت
- محدودية المال او التأمين
- محدودية الحصول على الرعاية الصحية
- قلق من اللقاح نفسه
- اخرى

6.5 إذا تم توفير لقاح لـ COVID-19 وقيل لك إنه سيحمي نصف (50%) الأشخاص الذين تلقوه ، فما مدى احتمالية تلقيك للتطعيم؟

- محتمل للغاية
- محتمل إلى حد ما
- ليس محتملًا ولا مستبعدًا
- غير محتمل إلى حد ما
- مستبعد للغاية

6.6 إذا تم توفير لقاح لـ COVID-19 وقيل لك أنه سيحمي (75%) من تلقوه ، فما مدى احتمالية تلقيك للتطعيم؟

- محتمل للغاية
- محتمل إلى حد ما
- ليس محتملًا ولا مستبعدًا
- غير محتمل إلى حد ما
- مستبعد للغاية

6.7 إذا تم توفير لقاح لـ COVID-19 وقيل لك أنه سيحمي 99% من تلقوه ، فما مدى احتمالية تلقيك للتطعيم؟

- محتمل للغاية
- محتمل إلى حد ما
- ليس محتملًا ولا مستبعدًا
- غير محتمل إلى حد ما
- مستبعد للغاية

6.8 الأشخاص الآخرون الذين يتم تطعيمهم ضد COVID-19 سيساعدون في السيطرة على الوباء

- أوافق بشدة
- موافق إلى حد ما
- لا أوافق ولا أختلف
- لا أوافق إلى حد ما
- أعارض بشدة

7.1 .اللقاحات مهمة للوقاية من الأمراض الخطيرة

- أوافق بشدة
- اوافق
- غير مؤكد
- غير موافق
- أعارض بشدة

7.2 قد يكون إعطاء أكثر من لقاح واحد في نفس الوقت غير آمن لصحة طفلي

- أوافق بشدة
- اوافق
- غير مؤكد
- غير موافق
- أعارض بشدة

7.3 الآثار الجانبية لمعظم اللقاحات تفوق الفوائد

- أوافق بشدة
- اوافق
- غير مؤكد
- غير موافق
- أعارض بشدة

7.4 أشعر بالقلق من أن الوتيرة المتسارعة لاختبار لقاح جديد لـ COVID-19 ستفشل في اكتشاف الآثار الجانبية أو الأخطار المحتملة.

- أوافق بشدة
- اوافق
- غير مؤكد
- غير موافق
- أعارض بشدة

7.5 اللقاح مهم لإنهاء وباء COVID-19

- أوافق بشدة
- اوافق
- غير مؤكد
- غير موافق
- أعارض بشدة

8.1 ما هو الحد الأدنى من الوقت الذي تستغرقه عملية الاختبار والذي من شأنه أن يجعلك تشعر بالراحة مع لقاح COVID-19؟

- ○ 3-6أشهر
- ○ 6أشهر إلى سنة
- ○ 1-2سنوات
- ○ بين 2 و 5 سنوات
- ○ أكثر من 5 سنوات

8.2 يرجى الترتيب من 1-5 إلى أي مدى توافق على العبارات التالية ، حيث 1 يرفض بشدة و 5 موافق بشدة

	1 ارفض بشده	2 ارفض	3 لا أوافق ولا ارفض	4 موافق	5 موافق بشده
أنا قلق من أن اللقاح نفسه سوف يعطيني COVID-19.	○	○	○	○	○
أفضل بناء المناعة من خلال التعرض لفرد مصاب بدلاً من تلقي اللقاح	○	○	○	○	○
من المرجح أن أحصل على اللقاح إذا لزم السفر دوليًا.	○	○	○	○	○
اعتقد ان مناعة القطيع كافية لحماية الجميع.	○	○	○	○	○
أنا قلق بشأن تكلفة لقاح .COVID- 19	○	○	○	○	○
أنا قلق من الآثار الجانبية للقاح بالنسبة لي.	○	○	○	○	○
أنا قلق من الآثار الجانبية للقاح للأطفال.	○	○	○	○	○
من المحتمل أن تكون الآثار الجانبية للقاح أسوأ من الإصابة بحد ذاتها	○	○	○	○	○
معرفة ان لقاح COVID-19 تم تطويره في أمريكا او اوروبا سيجعلني أشعر براحة أكبر عند تلقيه	○	○	○	○	○

معرفة أن لقاح COVID قد تم تطويره في مكان آخر غير أمريكا أو أوروبا سيجعلني أشعر براحة أكبر في تلقيه.	○	○	○	○	○

8.3 ما هو حجم مشكلة COVID-19 في قطر والعالم؟

- ليست مشكلة على الإطلاق
- تافهة مقارنة بالمشكلات الأخرى
- إلى حد ما من مشكلة
- مشكلة خطيرة أهم من معظم القضايا الأخرى
- أهم مشكلة تواجه العالم الآن

8.4 ما هو اللقاح التي تعتقد انه امن ؟

- لقاح اوكفورد-استرا زينيكا المنتج في بريطانيا
- لقاح فايزر المنتج في الولايات المتحدة الامريكية
- لا فرق بينهما

8.5 الرجاء الإجابة على الأسئلة التالية بكلماتك الخاصة: أكبر مخاوف لدي بشأن لقاح COVID-19 هو ...

<اكتب هنا>

8.6 الرجاء الإجابة على السؤال التالي بكلماتك الخاصة: ما الذي يجعلك أكثر راحة لفكرة تلقي لقاح لـ COVID-19؟

<اكتب هنا>
